# Comparing the implementation of team approaches for improving diabetes care in community health centers

**DOI:** 10.1186/s12913-014-0608-z

**Published:** 2014-12-03

**Authors:** Philip J Van der Wees, Mark W Friedberg, Elena Alcala Guzman, John Z Ayanian, Hector P Rodriguez

**Affiliations:** Department of Health Care Policy, Harvard Medical School, Boston, MA USA; Scientific Institute for Quality of Healthcare, Radboud University Medical Center, Nijmegen, the Netherlands; The RAND Corporation, Boston, MA USA; Division of General Medicine and Primary Care, Brigham and Women’s Hospital, Boston, MA USA; Community Health Partnership of Santa Clara County, San Jose, CA USA; Institute for Healthcare Policy and Innovation, University of Michigan, Ann Arbor, MI USA; Division of Health Policy and Management, University of California, Berkeley 50 University Hall, Room 245, Berkeley, CA 94720 USA

**Keywords:** Diabetes mellitus, Implementation, Community health centers, Organizational change

## Abstract

**Background:**

Patient panel management and community-based care management may be viable strategies for community health centers to improve the quality of diabetes care for vulnerable patient populations. The objective of our study was to clarify implementation processes and experiences of integrating office-based medical assistant (MA) panel management and community health worker (CHW) community-based management into routine care for diabetic patients.

**Methods:**

Mixed methods study with interviews and surveys of clinicians and staff participating in a study comparing the effectiveness of MA and CHW health coaching for improving diabetes care. Participants included 24 key informants in five role categories and 249 clinicians and staff survey respondents from 14 participating practices. We conducted thematic analyses of key informant interview transcripts to clarify implementation processes and describe barriers to integrating the new roles into practice. We surveyed clinicians and staff to assess differences in practice culture among intervention and control groups. We triangulated findings to identify concordant and disparate results across data sources.

**Results:**

Implementation processes and experiences varied considerably among the practices implementing CHW and MA team-based approaches, resulting in differences in the organization of health coaching and self-management support activities. Importantly, CHW and MA responsibilities converged over time to focus on health coaching of diabetic patients. MA health coaches experienced difficulty in allocating dedicated time due to other MA responsibilities that often crowded out time for diabetic patient health coaching. Time constraints also limited the personal introduction of patients to health coaches by clinicians. Participants highlighted the importance of a supportive team climate and proactive leadership as important enablers for MAs and CHWs to implement their health coaching responsibilities and also promoted professional growth.

**Conclusion:**

Implementation of team-based strategies to improve diabetes care for vulnerable populations was diverse, however all practices converged in their foci on health coaching roles of CHWs and MAs. Our study suggests that a flexible approach to implementing health coaching is more important than fidelity to rigid models that do not allow for variable allocation of responsibilities across team members. Clinicians play an instrumental role in supporting health coaches to grow into their new patient care responsibilities.

## Background

The high prevalence of diabetes is a major concern for health care costs and outcomes in the United States [1]. Many patients with diabetes do not receive optimal care, with large disparities in diabetes care documented for Latinos, Blacks and Asians in the United States [2]. Well-planned multi-component programs for the management of diabetes often require that the delivery of primary care be reorganized to more effectively provide self-management support to patients [3]. However, community health centers (CHCs) serving vulnerable patient populations face serious challenges with shortages of primary care clinicians [4]. Patient panel management and community-based care management may be viable strategies to improve diabetes care. Few studies, however, have assessed processes to introduce providers in new roles to improve diabetes care management in CHCs serving low-income, vulnerable patient populations.

Primary care teams are faced with managing high levels of heterogeneity in tasks and types of patients. The Institute of Medicine identified a set of core principles for high-value team-based health care: shared goals, clear roles, mutual trust, effective communication, and measurable processes and outcomes [5]. Several models have been developed and implemented to coordinate the activities of health care team members, for which team structure and processes are main components for stimulating teamwork [6]. Bodenheimer proposed a framework for high-performing primary care for achieving the triple aim of health reform—better health, improved patient experience, and more affordable costs [7].

Patient panel management comprises a set of tools and processes at the level of a primary care patient panel and entails a proactive approach to managing patient care that includes care by clinicians and follow-up activities by medical assistants (MA) [8]. The Teamlet Model of primary care, a small team comprised of a clinician and a MA as “health coach”, was introduced as an extension of the traditional clinician visit, by introducing visits with MAs to provide chronic disease self-management support [9,10]. Augmenting the panel management responsibilities of clinicians with MAs is an especially promising innovation for resource-constrained CHCs.

Community health workers (CHW) can play an important role in the outreach to underserved populations. They serve as bridges between their ethnic, cultural, or geographic communities and health care providers, and they engage their community to prevent diabetes and its complications through education, lifestyle change, self-management, and social support [11]. Previous research on CHW interventions for diabetes care demonstrates the potential for CHWs to improve clinical quality and patient care experiences [12].

The objective of our study was to clarify implementation processes and experiences of integrating office-based MA panel management and CHW community-based management into routine care for diabetic patients. To allow for a ‘real world’ implementation of the new roles of MAs and CHWs, clinic stakeholders were provided with significant latitude to integrate the new MA and CHW roles in their existing workflow. We posited that successful integration of the new roles into routine care for patients with diabetes would be linked to the baseline team functioning and the organizational capabilities of the participating CHCs [13,14]. We examined the early experiences of integrating office based panel management and community based management in routine primary care, and sought to identify implementation facilitators and barriers.

## Methods

### Study design and setting

A comparative effectiveness study provided the basis for our implementation study of 17 practice sites of six CHCs in three northern California counties. Practice sites were allocated to the MA intervention group (n = 3), CHW intervention group (n = 3), or control group (n = 11). The intervention sites were supported to implement the Teamlet Model [10] for panel management by MAs or community-based care management by CHWs. The MAs and CHWs participated in a two day training program tailored to their unique roles in diabetes care management, focusing on their roles in the work flow of managing the pre-visit, visit, post-visit, and between visits of diabetic patients. They were specifically trained in providing appropriate health education and information to patients, to offer counseling and social support, and in self-management and motivational interviewing strategies to stimulate lifestyle changes. After the initial training, technical assistance visits (n = 2-3) at each site were conducted by a quality improvement organization in order to support new staff and their care teams in implementing practice changes and modifying workflow to integrate new health coaching and self-management support roles. Table 1 delineates the distinctions between the MA and CHW roles. Participating CHCs received technical assistance to facilitate data collection and workflow. One intervention site (MA panel manager) and two control sites dropped out of the trial because of competing demands (n = 1) and clinical quality data submission problems (n = 2) during the first two months of the 12 month intervention period. Consequently, our key informant interviews with primary care clinicians (physicians and nurse-practitioners) and staff are limited to the 14 CHC sites fully participating in the project.

**Table 1:** **Activities of Medical Assistant (MA) and Community Health Worker (CHW)**

	**MA**	**CMW**
**Pre-visit**		
Discuss the patient case with the clinician	X	
Agenda setting with the patient	X	
Ordering routine services	X	X
History tracking	X	X
**During the visit**		
Document clinician findings	X	
Send electronic descriptions to pharmacy	X	
Write prescriptions for the clinician to sign	X	
**Post-visit**		
Discuss patients’ concerns	X	X
Recapitulate the advice given by the clinician	X	X
Set goals with the patient	X	X
Make sure that patients can navigate the system	X	X
**Between visits**		
Provide culturally appropriate health education and information	X	X
Assure that people with diabetes receive the services they need	X	X
Follow up via telephone	X	X
Offer informal counseling and social support		X
Provide information to families to support lifestyle changes		X
Build individual and community capacity		X
Make home visits to patients		X
Reach out into the community of patients		X

We conducted early implementation interviews of key informants from May-August 2012. This approach allowed for in-depth data collection to identify changes in care processes and assess facilitators and barriers to implementing the two strategies into routine practice during the early stages of implementation.

Six months prior to the implementation of the study, we conducted a clinician and staff survey to measure cultural aspects of the CHC sites, including care team functioning and organizational readiness for change. We used the respondent sample of clinicians and staff (n = 249) from the 14 CHC sites. The survey was administered to all primary care clinicians and staff and included 22 items related to care team functioning, quality emphasis, leadership readiness for change, team stability and norms, team harmony and inter-dependence, overload and chaos, staff readiness for change, general attitudes toward teamwork. These items are all from previously validated practice climate instruments, including the Team Diagnostic Survey (TDS) [15]; Attitudes Toward Health Care Team Scale [16]; Team Climate Inventory (TCI) [17]; Minimizing Error, Maximizing Outcome (MEMO) [18]; AHRQ TeamSTEPPS Teamwork Perceptions Questionnaire (AHRQ T-TPQ) [19]; TransforMed Clinician Staff Questionnaire (TransforMed CSQ) [20]; AHRQ Medical Office Survey on Patient Safety Culture [21]; and Organizational Readiness to Change Assessment (ORCA) [22]. Each item included multiple statements. An example of a statement related to care team functioning: “Everyone of your team is motivated to have the team succeed”. The statements were scored on a 5-point Likert scale ranging from “strongly disagree” to “strongly agree”.

In addition, each CHC practice site leader completed a survey of the clinics’ structural capabilities. Previous analyses showed that the availability of structural capabilities may benefit practices serving patients from socio-demographically vulnerable neighborhoods [14]. The structural capabilities survey included 34 items related to clinic characteristics falling in four domains: (1) patient assistance and reminders (e.g. assistance of patient self-management); (2) culture of quality (e.g. frequent meetings on quality performance; (3) enhanced access (e.g. multilingual clinicians; and (4) Electronic Health Records (EHR) (i.e. frequently used, multifunctional EHRs used during clinical care).

### Selection of participants

We invited clinicians and staff from each of the 14 CHC sites by email to participate in a 30-60 minute key informant telephone interview and followed up with non-respondents by phone. For interviews, we used quota sampling [23] to select key informants from each of the following five roles: practice coordinator, clinician, medical assistant, community health worker, and other roles (nurse/allied health professional). We aimed to interview all MA panel managers and CHWs across the five intervention sites, as well as a practice coordinators and clinicians from each of the intervention sites. Key informants at control sites were selected for invitation with the aim of interviewing at least one key informant for each of the eleven control sites, continuing interviews until the themes reported reached saturation.

### Ethics statement

The study was approved by the Institutional Review Board of the University of California Los Angeles (IRB #10-000596). All key informants received written information about the purpose and procedure of the interviews. Interviews were scheduled after the key informants confirmed their participation via email. Final oral informed consent for recording and transcribing the interviews was obtained from all participants at the beginning of each interview based on a standardized consent script, which was approved by the Institutional Review Board of the University of California Los Angeles. The oral consent was documented by the interviewer prior to the recording of the interview. Reason for obtaining final oral consent at the beginning of the interview was to ensure that the key informants understood the procedure and to allow them to ask any questions about their rights and protection of privacy.

### Interviews

All interviews were conducted by telephone by one of five interviewers and were audio recorded. Interview guides were used to guide the semi-structured interviews in assessing implementation processes and diabetes care quality improvement activities. The aim of the interviews was to learn about changes in the management of diabetes care at the CHC site, and perceived barriers and facilitators that occurred during the early phases of the intervention period. Although the control sites were not encouraged to implement any changes, it is possible that the practices engaged in other changes that impact diabetes care management. We wanted to identify possible changes in the control clinics to understand secular trends in diabetes care management among the CHC sites.

The interview guides began with general questions about the key informant’s background and his/her position, followed by specific questions about the specific role of the interviewee in managing patients with diabetes, composition of their care team, and the use of specific strategies to better coordinate and integrate the care of patients with diabetes. The interview guides were tailored to the specific role of each of the five key informant categories. We asked practice coordinators about strategies at the clinic level in improving the care for patients with diabetes; clinicians were asked about their responsibility in improving the care for patients with diabetes, other health professionals were asked about their role in the multidisciplinary team. An additional module was used for the MA and CHW roles to assess the implementation of health coaching activities, and tailoring of care to cultural diversity of patients.

### Data analysis

Interviews were transcribed verbatim, cleaned, de-identified, and entered into Atlas.ti qualitative software for analysis. We used a qualitative content analysis with a directed approach [24]. A priori themes were derived from behavioral change of professionals [25], team functioning [26,27], structural capabilities at the organizational level [14,28], and cultural context and competence [29-32]. A framework of codes was developed by PJW and HPR, and checked for consistency by MWF and JZA. PJW and DL independently coded one transcript for each of the key informant roles, to allow inclusion of emergent codes from the interviews themselves. A log was maintained of emerging codes and used for adapting the coded framework. The adapted framework was reviewed for consistency by MWF and JZA. The final list of codes was applied to all interviews by PJW and DL for each of the key informant roles. Coding discrepancies were reconciled during regular team meetings.

We conducted a thematic analysis of each of the key informant roles to identify diabetes care improvement strategies as well as perceived barriers and facilitators to implementing these strategies. Coded text was tabulated by theme, clinic site and key informant role to identify patterns within and between clinics.

For the clinician and staff survey, we computed site-level composite scores (range 0-100) to characterize the clinic’s staff relations, quality improvement orientation, manager readiness for change, staff readiness for change, teamwork attitude and clinic workload. Following Friedberg et al. [14], the following structural capabilities were assessed: patient assistance and reminders, electronic health records, culture of quality, and other practice characteristics. We examined differences among intervention and control CHC sites using t-tests for continuous variables and chi-square tests for categorical variables. We then triangulated results from the clinician and staff survey, practice leader survey, and key informant interviews to identify concordant and disparate results across the data sources.

## Results

Interviews were conducted with key informants from all (n = 5) intervention practices and seven of the nine control practices fully participating in the trial. A total of twenty-four key informants participated (responsive rate = 86%); 13 from intervention clinics and 11 from control clinics. Table 2 describes the distribution of the key informant roles among the clinics. All clinics provided general primary care services to low-income families and individuals, with large populations of Latino and Asian patients. The average number of clinicians was 4.8 per clinic (range, 2-9). Table 3 describes the setting, patient population and characteristics of health coaching in the intervention clinics.

**Table 2:** **Participating key informants**

**Key informant role**	**Intervention (5 clinics) N**	**Control (7 clinics) N**	**Total (12 clinics) N**
Practice leader (coordinator, medical director)	3	2	5
Clinician (physician, nurse practitioner)	4	4	8
Medical assistant	3	3	6
Community health worker	3	0	3
Other (nutritionist and registered nurse)	0	2	2
	13	11	24

**Table 3:** **Setting, patient population and characteristics of health coaching in the intervention clinics**

**Clinic**	**Setting**	**Provider organization**	**Health coach**	**Team composition**	**Panel size (Overall diabetics at clinic)**	**Main patient population**	**Workflow**
1.	Urban	2 clinics with ~5 clinicians serving low-income families	MA	Team of 2 clinicians and 2 MA	119 (139)	Latino	MA panel management based on the Teamlet Model^1^. No home visits. Combining regular MA work with health coaching. MA sees 4 patients per day for health coaching on alternate days.
2.	Urban	7 clinics with ~50 clinicians serving low-income families	MA	Team of 6 clinicians and 4 MA.	NS (367)	Recent Chinese immigrants	MA works on weekly rotating schedule as health coach. Sees ~12 patients per day typically in post-visits to clinician. No home visits.
3.	Small community	7 clinics with ~40 clinicians serving low-income families.	CHW	Team of 2 clinicians and 1 CHW	118 (334)	Latino	CHW works mainly office-based via panel management in Teamlet Model. Sees 6-8 patients per day.
4.	Small community	2 clinics with ~5 clinicians serving low-income families.	CHW	Team of 3 clinicians and 2 CHW	137 (143)	Latino	CHW does office-based visits and post-visits based on Teamlet Model. Started small-scale home visits, planning 3-4 joint visits per day by 2 CHW.
5.	Suburban	7 clinics with ~40 clinicians serving low-income families.	CHW	Team of 3 clinicians and 1CHW.	84 (377)	Latino	CHW works community-based with home visits of 25-30 minutes during 4 days per week. One day office-based for follow-up phone calls. Separate from clinic workflow.

MA: Medical Assistant; CHW: Community Health Worker; NS: No Specified Patient Panel.

^1^Teamlet Model refers to a small team comprised of a clinician and a MA as “health coach” as an extension of the traditional clinician visit, by introducing visits with MAs to provide chronic disease self-management support.

Summary statistics for the care team functioning and organizational readiness for change composites are presented in Table 4. Mean values in the intervention and control group ranged from 62.7 to 70 (on a 0-100 scale) for staff relationships, quality improvement, manager readiness for change, and staff readiness for change. Teamwork attitude scored an overall mean of 55, and clinics scored workload with an overall mean of 43. None of the six composite measures differed significantly between the intervention and control clinics.

**Table 4:** **Care Team Functioning and Organizational Readiness for Change at Baseline**

Composite measure*	**Intervention mean (Range)**	**Control mean (Range)**	**Overall mean (Range)**
Staff relationships	64.8 (46.9 to 78.2)	65.7 (56 to 81.7)	65.2 (46.9 to 81.7)
Quality improvement	66.6 (49.4 to 76.5)	62.7 (49.1 to 76)	65.2 (49.1 to 76.5)
Manager readiness for change	63.8 (42.5 to 77.5)	63.9 (38 to 80.8)	63.9 (38 to 80.8)
Staff readiness for change	70.0 (60.4 to 81.2)	67.6 (52.8 to 74.6)	69.2 (52.8 to 81.3)
Teamwork attitude	55.0 (50.0-60.0)	55.0 (52.5-57.5)	55.0 (50.0-60.0)
Clinic workload	44.9 (33.6 to 60.1)	41.7 (30.4 to 76)	43 (30.4 to 60.1)

*Composite scores (range 0-100) based on clinician/staff survey prior to the intervention to measure cultural aspects of the control and intervention sites. None of the composite measures differed significantly between the intervention and control clinics based on *t*-test statistics.

The structural capabilities of the clinics for reminder systems, registries and language services survey are presented in Table 5. Four out of five intervention clinics had reminder systems for monitoring of hemoglobin, cholesterol, eye exams and nephropathy (none were electronic); and six out of eight control clinics had such reminder systems in place. Registries for monitoring and benchmarking patients with diabetes who were out of target range for hemoglobin level were available in three intervention clinics and two control clinics.

**Table 5:** **Structural capabilities at baseline**

**Structural capability** ^*^	**Intervention (n = 5)**	**Control (n = 8)†**
*Checklist or flow-sheet for*:		
HbA1c testing	4/5	6/8
Cholesterol testing	1/5	6/8
Eye examination	4/5	6/8
Nephropathy monitoring	4/5	6/8
*On-site registry out of target range for*:		
Laboratory values	3/5	2/8
Physical findings (BP, BMI)	3/5	2/8
*On site registry for patients overdue for*:		
Screening services	5/5	6/8
Diabetes services	3/5	4/8
Other chronic disease services	0/4	3/8
*Shared communication*:		
HbA1c testing	2/4	2/8
Cholesterol testing	2/4	2/8
Eye examination	2/4	2/8
Nephropathy monitoring	2/4	1/8

MA denotes Medical Assistant; CHW denotes Community Health Worker.

^*^Proportions of available capabilities are listed for the two arms of the intervention clinics (office-based panel management and community-based management) and the control clinics. Due to low sample sizes, statistical testing for differences between intervention and control was not performed.

†One control group did not fill out the questionnaire.

The thematic analysis of the key informant data identified two main domains associated with deeper implementation of the new care team roles for diabetes care: health coaching responsibilities and practice culture. Within the health coaching domain, we organized a subtheme to understand cultural adaptations to standard diabetes self-management approaches used by CHWs and MAs. Within the practice culture domain, we distinguished the following four subthemes: team composition, care team functioning, structural capabilities, and leadership. Table 6 summarizes the themes and major findings in the qualitative analysis and we elaborate on these findings in the following paragraphs. Triangulation of key informant interview and practice climate survey data confirmed the four subthemes in the practice culture domain. Structural capabilities, as ascertained via the survey, may have supported the implementation of health coaching activities regardless of the model used with office-based patient MA panel management or community-based care by CHW. There were no differences between clinics at baseline in terms of care team functioning and organizational readiness for change in the quantitative analysis. This is congruent with our qualitative analysis revealing similar facilitators and barriers to care team implementation for diabetic patients across the clinics. Below we elaborate on the role of health coaching activities and practice culture as they relate to aiding implementation of new CHW and MA roles and responsibilities for supporting diabetes care management.

**Table 6:** **Summary of themes and major findings in the qualitative analysis**

**Health coaching**	**Practice culture**
Flexibility and latitude of health care teams in panel management and home visits	Team composition of dedicated MA/CHW with collaborating clinicians (vs. rotating MA/CHW)
Cultural adaptation to target population	Care teams supported by practice climates conducive to facilitating the transition of diabetes self-management support responsibilities to CHW/MAs, warm handoff by clinician and acceptance of patients
	Structural capabilities to stimulate monitoring of diabetes care process and outcomes
	Active support of leadership in MA/CHW health coaching

### Health coaching

The MAs and CHWs in the intervention practices considered health coaching as the most essential aspect of their new roles. The key informants in the control clinics also found health coaching to be a very important skill, but they all acknowledged that they had not successfully implemented health coaching responsibilities in their clinics. Two control clinics implemented health coaching responsibilities for staff on a small scale, but difficulties in integrating the activities into the routine workflow of busy practices halted these efforts.

MAs and CHWs performed similar duties as health coaches for patients with diabetes, including patient education, goal setting, action planning, and evaluation. All participants emphasized the importance of self-management in improving diabetes care and patient responsibility to control their own health, and they reflected on the valuable training they received to implement the new team approaches. One MA reflected, “As we were taught to do in training, forcing is not a good way to achieve the goals. Most of the time, I will let patients make the decision”.

#### Cultural adaptation

The MA and CHW health coaches in the intervention clinics were aware of the importance of tailoring care to individual patients’ needs. They described considerable efforts in building rapport with their patients and ensuring they were addressing the individual needs of their patients. All health coaches were bilingual in English and either Spanish or Mandarin, and were able to communicate with most patients in their primary language. The majority of the intervention and control clinics provided information material that was translated in at least one language (Spanish or Mandarin). The absence of translated material was considered an important barrier for health coaching. “If health education materials were translated into Spanish, it would be a lot easier” mentioned one MA.

Most, but not all, MA and CHW health coaches had similar race/ethnicity as the majority of their patients. This was considered an advantage, as one CHW explained, “With me it’s just like someone from my background it’s easier to identify healthy meals that are acceptable”. The MA and CHW health coaches also played an important role in translating for clinicians who did not speak the primary language of their patients.

### Practice culture

#### Team model

Table 3 describes characteristics of the primary care team models used by the practices. Three intervention clinics implemented health coaching activities using CHWs. Two of these clinics appointed a single full-time CHW to fulfill the new role for the practice. One clinic appointed a part-time CHW and recently trained a second CHW to support in the health coaching activities. Two of the three interviewed CHWs had professional training as MAs, and the third CHW had experiences as a social services outreach worker.

Care teams in the intervention clinics consisted of the clinician, the regular MA, and the new MA/CHW health coach. The MA and CHW health coaches reported referring patients to nutritionists on a regular basis. Nurses were not mentioned in a core team role and were generally employed in primary care clinical roles in the CHCs. The control clinics typically organized their care around the clinician and regular MA, where the clinician, who was solely responsible for patient education, provided limited health coaching.

The care team models varied considerably among the three CHW intervention practices. One practice implemented routine home visits by the CHW to support self-management, while another practice had only recently began to implement home visits for diabetic patients with abnormal clinical values of hemoglobin levels. The CHW in the third practice faced reluctance of patients towards home visits. The health coaching activities of the CHW in this practice were office-based, using the teamlet approach with a panel of 140 diabetic patients. Practice informants indicated that gender discordance may have been an issue: the CHW was male, and female patients may have felt uncomfortable with a man entering their home. As one clinician indicated, “We thought maybe it’s a male versus a female, and people aren’t quite as trusting with a male coming in”.

The two intervention sites that implemented MA panel management also differed in their approaches to implementing the new role. One clinic incorporated the Teamlet approach using two part-time MA health coaches sharing a panel of 120 patients with diabetes. Another clinic used four MA health coaches on a weekly rotating basis. For example, one MA served as health coach for one week and then had three weeks on other duties, while the health coaching responsibility was rotated to other staff. The control clinics’ primary care teams consisted of clinicians working with a dedicated or ‘floating’ MA to assist with logistics and the provision of some routine services. One control clinic recently started implementing the Teamlet model on a small scale, but efforts were not specific to patients with diabetes. None of the control clinics used CHWs in primary care teams.

The practice that implemented health coaching by an MA using a weekly rotating schedule initially rotated MA staff daily, but changed to a weekly rotation because continually switching was confusing to other team members. The interview informants at this CHC site varied in their responses about whether the rotating schedule was optimal. One clinician indicated, “I would have probably wanted to have just one staff and a backup devoted to this position, so as not to have so much change. Since we rolled it out rather quickly, there wasn’t that kind of option”; while a contrasting response from a MA from this clinic was, “This kind of [rotating] arrangement can help me to refresh my knowledge on other clinical skills and also to refresh internal medicine skills…we all like the rotations”.

The MA panel management intervention clinics were all challenged by the fact that MAs did not have enough dedicated time to conduct health coaching activities because regular MA responsibilities often “crowded out” their less time-sensitive health coaching responsibilities. The hectic schedule of regular MA duties may interfere with the health coaching, especially on days when demand is high and staffing is low. As one MA health coach indicated, “Sometimes clinic demands interfere- like we need to help out the clinic flow, or when we are short on manpower. Sometimes, a clinic colleague can’t make it that day; then we need to follow doctors to cover for the regular MA jobs”. Two of the three CHW clinics appointed health coaches in a full time position with a unique and separated role. Relative to the MAs, the CHW health coaches were more satisfied with their dedicated time, which allowed them to build expertise and become specialists in their job without feeling peer pressure.

A consistent finding throughout the interviews was that over time, CHW and MA responsibilities in supporting patient care converged. Although the MAs and CHWs were trained to fulfill different activities of office-based panel management vs. home visits respectively (see Table 1), their ultimate approach in health coaching activities was comparable.

#### Care team functioning

Responses from both sets of intervention clinics (MA and CHW) emphasized the importance of support from the collaborating clinician and practice leader. A supportive team climate that enabled MAs and CHWs to take responsibility for their health coaching activities was mentioned as an essential component to grow in their new role and to gain trust from patients. Practices with implementation of the new CHW and MA roles, tended to have practice leaders who were described by informants as committed to organizing the project and supporting the MA and CHW by providing the conditions for them to be effective in their new roles. Clinicians were described as being generally supportive of the new health coach roles of MAs and CHWs, although some took a more active role than others. Participants reported a lack of consistent introductions of patients to health coaches by clinicians due to time constraints. “If the clinician introduces the health coach correctly and does a warm handoff, the patient is actually very willing to listen to the health coach. But the problem is getting clinicians to do that warm handoff.” The MA and CHW health coaches all expressed great pride in their new positions, indicating that they felt appreciated by members of the primary care team as well as by patients. One MA commented: “I’ve been putting myself in the provider’s shoes—and the patient’s shoes—and it’s been very, very helpful for me to understand and …be the bridge between the provider and the patient. The patients have shown very good results and [had] very good comments about this …”

#### Structural capabilities

Electronic health records (EHR) were used, to some extent, by all intervention and control clinics. In two intervention practices, EHRs were recently introduced. Key informants in the intervention clinics were generally positive about EHR implementation. Reminder systems and registries were implemented in the majority of the intervention clinics (Table 4), although none of the clinics used an electronic reminder system and only one intervention clinic used an electronic registry. Therefore, structural capabilities showed considerable room for development, both in the intervention clinics and the control clinics. Study personnel provided a computer-based mapping system to the intervention clinics, which was designed primarily to assist CHWs in finding nearby health resources for patients. The MA and CHW health coaches were enthusiastic about this tool to guide patients in using community-based activities, such as recreational facilities for exercise or healthy and inexpensive food stores.

#### Leadership

The active support of practice leadership was considered important by the MA and CHW health coaches to execute their new roles effectively. Practice coordinators and clinicians allowed staff autonomy and flexibility to fulfill their new roles. As one MA indicated, “My clinic coordinator is very protective about my time. When someone wants to assign me something else or whatever she’s very, very good. I think that that has a lot to do, also, with the success of this project is that you get the support from your supervisor and also the clinicians” The MA and CHW health coaches felt supported by the practice leadership, although they were largely unaware of the level of involvement of executive leadership. The practice leaders and clinicians were more aware of the role of executive leadership, which always delegated the responsibility to coordinate and supervise the primary care team. One of the main issues for continuing management support for the CHW and MA role in diabetes care management was the sustainability of the model after project funding ended. Overall, key informants in the intervention clinics were moderately optimistic about the sustainability of the health coaching approach to diabetes care management, but responded that concrete evidence for the effectiveness and efficiency of the MA and CHW health coaches would be necessary to convince the management to allocate sufficient resources to maintain the team approach over time.

## Discussion

Our results show how real world implementation of health coaching strategies to improve diabetes care management differed considerably between clinics during the early implementation. The integration of these strategies into routine practice required flexibility and latitude of primary care teams. In contrast, the health coaching roles of CHWs and MAs converged over time despite the emphasized role differences in their training. MAs found it more difficult than CHWs to allocate dedicated time due to their regular MA responsibilities. For the practice leadership the implementation of MA health coaches seems to be more feasible in combining their new role with regular MA activities.

Bodenheimer [7] identified four foundational elements for high-performing health care: engaged leadership, data-driven improvement, empanelment, and team-based care. Our findings derived from both quantitative and qualitative data confirm the importance of these elements. A supportive team climate with engaged leadership enabled MAs and CHWs to take responsibility for their health coaching activities. In addition, structural capabilities were important for data-driven improvement for both MA panel management and CHW community-based care.

Implementation fidelity is the degree to which programs are implemented as intended by the program developers [33,34]. The results of our study can be interpreted in several ways. One way is that fidelity of implementation of the MA vs. CHW roles was low (Table 1). Only one of the three CHW clinics implemented routine home visits and only one of the two MA clinics used the Teamlet model of primary care to implement MA responsibilities that were distinctive aspects of the respective roles. Intervention practices implemented the new roles in ways that provided latitude for tailored integration of the new MA and CHW responsibilities into routine clinical care processes rather than creating a new silo. The varying infrastructure of the clinics, the low-income Latino and Asian patients they serve, and the resources available in their communities required different approaches to implement the new roles; thus enhancing acceptability and adoption of clinicians and patients. Although an adaptive approach creates analytic complexities in the execution of a comparative effectiveness research study, successful implementation of health coaching responsibilities was contingent on flexibility and discretion in practice implementation.

A consistent finding in our interviews of MA and CHW health coaches was the importance of a supportive team climate, allowing them to take responsibility for their health coaching activities and to gain trust from patients. This supportive team climate was both related to engaged clinic leadership as well as by warm handoffs clinicians to signal trust in team members’ abilities to patients. Informants attributed the successful implementation of health coaching activities of the MAs and CHWs to a supportive team climate that enabled them to have flexibility and discretion in executing their new roles. An important barrier for fulfilling their new responsibility was the lack of personal introduction of health coaches to patients by clinicians, which was attributed to time constraints in busy primary care practices. Dedicated time for clinicians to support the implementation of the Teamlet model of primary care, appropriate timing of health coaching sessions, and structured communication between health coaches and clinicians is likely needed to manage these workflow issues [35]. Early experiments implementing the Teamlet model show that some clinic staff members are not interested in health coaching, a role requiring a high degree of empathy, communication skills and ability to work in partnership with patients and clinicians [35,36]. This raises the question whether clinics should use multiple (part-time) health coaches paired with clinicians, versus one or two dedicated (full-time) health coaches in partnership with multiple providers. Having a “back-up” was perceived as advantageous, although the part-time nature of the position made it more difficult to ensure dedicated time for the MAs to conduct health coaching responsibilities, as opposed to the more exclusively dedicated role of the CHW health coaches.

Providing culturally competent care to low-income safety net patients involves tailoring care to meet patients’ social, cultural and linguistic needs [30,31]. Although all health coaches were aware of the importance of tailoring their care to individual patients, they considered a personalized approach more important than specific tailoring of their activities to the race/ethnicity of their patients. This may partly be due to the fact that most health coaches had a similar race/ethnicity as the majority of their patients. However, the health coaches responded to use the same approach across race/ethnicity of their patients. Additional professional education may enhance their cultural competence by understanding attitudes such as stereotyping, multifactorial causes of health disparities, and communication skills [37].

Structural capabilities aimed at the monitoring of health outcomes were available in most clinics, but there was also substantial room for improvement in the adoption and use of these capabilities. Our early implementation interviews did not allow for in-depth evaluation of perceived improvement of health outcomes. Nevertheless, our key informants expected a positive impact of their coaching activities on the health of their patients. They emphasized that such evidence would be necessary for sustaining their team-based health coaching and self-management support efforts for diabetic patients.

## Conclusion

In conclusion, the early implementation of MA and CHW care team approaches for improving diabetes care highlights the flexibility and discretion required of primary care teams for integrating new responsibilities into routine practice. Time constraints limited consistent introduction of health coaches to patients by clinicians and compromised dedicated time for health coaching activities. Allowing flexibility in approaches may deviate from the originally intended models, but promote acceptance and adaptation of innovative strategies to improve diabetes care. Care team functioning, organizational readiness for change, and structural capabilities showed room for improvement, emphasizing the need of flexibility. When practices face organizational challenges, local circumstances appear to drive solutions to address these challenges. The implementation of health coaching should be tailored to the needs of clinics and patient populations, and a flexible approach seems to be more important than fidelity to rigid models that have not been implemented in routine settings. Clinicians have a key position in supporting health coaches to grow in their roles and new professional responsibilities.
